# Hepatitis E, Central African Republic

**DOI:** 10.3201/eid1404.070833

**Published:** 2008-04

**Authors:** Josep M. Escribà, Emmanuel Nakoune, Carlos Recio, Péguy-Martial Massamba, Marcelle Diane Matsika-Claquin, Charles Goumba, Angela M.C. Rose, Elisabeth Nicand, Elsa García, Cornelia Leklegban, Boniface Koffi

**Affiliations:** * Médecins sans Frontières, Barcelona, Spain; † Institute Pasteur of Bangui, Bangui, Central African Republic; ‡ Ministry of Health, Bangui, Central African Republic; § University of the West Indies, Bridgetown, Barbados; ¶ National Reference Centre for Enterically Transmitted Hepatitis, Val de Grâce, Paris, France; 1 Current affiliation: Catalan Institute of Health, Barcelona, Spain.

**Keywords:** Hepatitis E virus, disease outbreaks, pregnant women, Central African Republic, letter

## Abstract

Hepatitis E, Central African Republic

**To the Editor**: Outbreaks of hepatitis E virus (HEV) have been documented in many geographic regions and nonindustrialized countries (*1**–**3*); they have been primarily associated with fecal contamination of drinking water (*4*). In the Central African Republic (CAR), economic indicators (CAR ranks 172/177 countries on the 2006 United Nations Development Program Human Development Index), political instability, geographic situation, a deteriorating health network, and a very poor epidemiologic surveillance system all contribute to the country’s epidemic susceptibility.

In July 2002, Ministry of Health (MoH) and Médecins sans Frontières (MSF) teams working in the Begoua Commune Health Center, north of CAR’s capital Bangui, reported an increased number of patients from the Yembi I neighborhood who were showing signs of jaundice and extreme fatigue.

Patients suspected of having hepatitis E were defined as those with clinical jaundice (yellow discoloration of the sclera) and symptoms of malaise, anorexia, abdominal pain, arthralgia, and fever. Confirmed cases were those in which patients’ serum samples were positive for HEV immunoglobulin (Ig) M or IgG.

Initially, 16 pairs of serum and stool samples were collected from jaundiced patients. Fecal samples were stored at –20°C and sent to the National Reference Center of Enterically Transmitted Hepatitis, Hospital Val de Grâce (Paris, France) for HEV marker testing; serum samples were tested at the Bangui Pasteur Institute for yellow fewer (YF) IgM by MAC-ELISA.

The HEV epidemic was confirmed by the detection of HEV markers: HEV IgG (Enzyme Immuno Assay, HEV, Abbott Laboratories, Abbott Park, IL, USA), HEV IgM (Abbott Laboratories), amplification of RNA (*5*), and the absence of YF IgM. The HEV genome was detected in 4 of the fecal samples. Genotyping and sequencing showed that one of these was genotype 1, prevalent in Africa; the others were related to genotype 2 (Mexico-like) (GenBank accession nos. DQ151640, DQ151640) (*5**,**6*).

Data suggest that the epidemic began in the Yembi I neighborhood, then spread to the rest of the Begoua commune and finally to Bangui or surrounding areas (Figure). Of 715 suspected HEV case-patients recorded in the MSF hospital between July 22 and October 25, 2002, 552 (77%) lived in the Begoua commune (271 in the Yembi I neighborhood). The attack rate for the Begoua commune (20,080 inhabitants) was 2.7%. Of 351 suspected case-patients serologically tested for IgG and IgM anti-HEV antibodies, 222 (63%) had IgM antibodies, including 5/16 pregnant women (2.3% of all confirmed cases). Most patients reported jaundice (97.5%) and choluria (95.1%);other reported symptoms were nausea and vomiting (37.9%), dyspepsia (28.3%), and hepatomegaly and/or splenomegaly (26.4%). Four of the confirmed case-patients died, a case-fatality ratio (CFR) of 1.8%; one was a pregnant woman (CFR 20% for pregnant women group).

**Figure:**
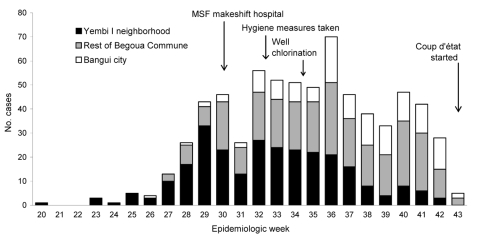
Suspected cases of hepatitis E virus in Begoua, Central African Republic, by neighborhood, weeks 20–43, 2002. MSF, Médecins san Frontières.

No significant differences were found among confirmed case-patients by sex or age-group. Seventy-seven (34.6%) had relatives with suspected HEV, and 163 (73.5%) had drunk untreated water from their own wells.

These epidemiologic findings suggest the water-borne nature of this outbreak. Environmental testing of water from 2 wells (before chlorination was implemented) showed the water to be unsafe to drink (i.e., heat-resistant coliforms and aerobic bacteria were present) (*7*).

The outbreak was not surprising because a 1995 survey in Bangui showed anti-HEV antibodies in 24% of patients tested (*8*), indicating endemic HEV. Our results for IgG-positive patients were similar (23.2% in men and 20.1% in women). As demonstrated during other outbreaks (*3*), we found no significant difference between the distribution of HEV-positive patients by age or sex, although most patients were males (58%) and young adults (71% of ages 14–45 years).

The observed CFR was similar to that in other reported HEV outbreaks, in which CFR varied from 1% to 4% (*9**,**10*), but it was as high as >30% in pregnant women (*9*). Deliveries during pregnancy months 6–8 in this outbreak highlight the need for close surveillance of pregnant women affected by this disease.

We recommended application of preventive measures, including water disinfection, safe disposal of excreta, community health education, and the strengthening of case management and disease surveillance. For the CAR, free access to a safe water supply and drugs was the only way to achieve these goals.

The number of HEV cases in the Yembi I neighborhood declined after the crisis team implemented hygienic and chlorination measures in the district, although the number of cases remained constant in other neighborhoods of the commune (Figure). Definite conclusions cannot be drawn from this finding. First, the MSF hospital was within the Begoua commune. Thus, patients from the rest of Bangui (outside the commune) only started arriving at the center for treatment after hearing about the hospital through broadcast messages. Second, a military coup d’état during epidemiologic week 43 prevented us from conducting further surveillance.

Our results agree with international data on HEV outbreaks in other nonindustrialized countries. However, studies to improve our understanding of this epidemic and to identify the main risk factors involved would be beneficial.
